# A Model Study on Raw Material Chemical Composition to Predict Sinter Quality Based on GA-RNN

**DOI:** 10.1155/2022/3343427

**Published:** 2022-04-12

**Authors:** Yifan Li, Qunwei Zhang, Yi Zhu, Aimin Yang, Weixing Liu, Xinfeng Zhao, Xinying Ren, Shilong Feng, Zezheng Li

**Affiliations:** ^1^Hebei Engineering Research Center of Iron Ore Optimization and Iron Pre-process Intelligence, North China University of Science and Technology, Tangshan, Hebei, China; ^2^Hebei Key Laboratory of Data Science and Application, North China University of Science and Technology, Tangshan, Hebei, China; ^3^The Key Laboratory of Engineering Computing in Tangshan City, North China University of Science and Technology, Tangshan, Hebei, China; ^4^College of Metallurgy and Energy, North China University of Science and Technology, Tangshan, Hebei, China; ^5^Tangshan Intelligent Industry and Image Processing Technology Innovation Center, North China University of Science and Technology, Tangshan, Hebei, China; ^6^College of Science, North China University of Science and Technology, Tangshan, Hebei, China; ^7^College of Yi Sheng, North China University of Science and Technology, Tangshan, Hebei, China

## Abstract

The quality control process for sintered ore is cumbersome and time- and money-consuming. When the assay results come out and the ratios are found to be faulty, the ratios cannot be changed in time, which will produce sintered ore of substandard quality, resulting in a waste of resources and environmental pollution. For the problem of lagging sinter detection results, Long Short-Term Memory and Genetic Algorithm-Recurrent Neural Networks prediction algorithms were used for comparative analysis, and the article used GA-RNN quality prediction model for prediction. Through correlation analysis, the chemical composition of the sintered raw material was determined as the input parameter and the physical and metallurgical properties of the sintered ore were determined as the output parameters, thus successfully establishing a GA-RNN-based sinter quality prediction model. Based on 150 sets of original data, 105 sets of data were selected as the training sample set and 45 sets of data were selected as the test sample set. The results obtained were compared to the real value with an average prediction error of 1.24% for the drum index, 0.92% for the low-temperature reduction chalking index (RDI), 0.95% for the reduction index (RI), 0.40% for the load softening temperature T_10%_, and 0.43% for the load softening temperature T_40%_, with all within the running time thresholds. The study of this model enables the prediction of the quality of sintered ore prior to sintering, thus improving the yield of sintered ore, increasing corporate efficiency, saving energy, and reducing environmental pollution.

## 1. Introduction

The increasing demand for steel has contributed to the rapid development of the steel industry. For blast furnace smelting, the main source of raw material is sinter ore, the quality of which has a direct impact on the quality of the iron obtained after this process, so, for environmental protection and blast furnace smelting, it is particularly crucial to improve the quality of sinter ore. The quality inspection process for production sintering is cumbersome and time- and money-consuming and the test results are delayed. When the assay results come out and the ratios are found to be faulty, the ratios cannot be changed in time, which results in the production of sintered ore of substandard quality, resulting in a waste of resources and environmental pollution. Therefore, predicting the quality of sintered ore before sintering in order to optimize the sintering process and control the quality of sintered ore is very important for blast furnace smelting as well as for environmental protection [1–10].

In the exploration of sinter quality prediction, Han developed a LIBS-based sinter alkalinity analysis technique based on the characteristics of sinter ore samples to achieve rapid detection of sinter alkalinity [11]. Chen et al. used the ironmaking principle, material balance principle, and expert knowledge as the theoretical basis to develop a sinter-blast furnace ironmaking whole process furnace charge structure intelligent optimization system [12]. Xiang Xiaoping designed a sintering process optimization and transformation plan to improve quality and reduce consumption. Through the “multipoint sectional” water refilling transformation, low negative pressure ignition transformation, bottom material grading fabric transformation, single roll Sandwich funnel optimization, and other measures, the sintering production cost was reduced and the quality of sintered ore production was steadily improved [13]. Based on the deep excavation of the microstructure of the mineral phase, Liang et al. identified the dependence of the sintered mineral phase characteristics on the metallurgical properties [14]. Yao et al. studied the equilibrium phase composition of sintered ores with different aluminium contents and the effect on the metallurgical properties of sintered ores using sample analysis by means of mineral phase microscopy, XRD, SEM, and EDS [15]. Zhang et al. used FactSage 7.1 thermodynamic software to simulate and analyse the effect of MgO on the liquid phase formation properties of sintered ore and investigated the effect of MgO on the liquid phase formation properties and microstructural properties of sintered ore [16]. The above research has laid the foundation for intelligent prediction of sintered ores.

With the development of deep learning and the improvement of blast furnace massification and smelting technology, deep network algorithms are being used in sinter quality prediction. Zhang et al. made a general analysis of the sintering process from the perspective of the operating characteristics of the auxiliary materials. A BP neural network algorithm with a quantitative term and variable learning rate was used to develop a sinter quality prediction model [17]. Wang et al. used online prediction of sinter quality based on Elman neural network. The experimental results showed that the online prediction significantly reduced the material conditioning time, improved the sintering process quality, and predicted the results more accurately [18]. Liu et al. established static BP neural network and dynamic Elman recurrent neural network for sinter quality prediction, respectively. Simulation experiments show that the prediction accuracy of the static BPNN prediction model built by applying industrial data training is higher than that of the Elman RNN model [19]. Li et al. proposed an intelligent integrated prediction model for the quality of cascade structure sinter ore, which was predicted by using a time series integrated prediction algorithm based on T-S fuzzy fusion and an information entropy integrated prediction model based on BP neural network with least squares support vector machine hierarchically, respectively. The sintered ore iron grade, alkalinity and drum index were effectively predicted [20].

In summary, in the exploration of predicting the quality of sintered ore, related scholars have made some progress in predicting the alkalinity *R* and drum index of sintered ore. However, few studies have been conducted to predict the physical and metallurgical properties of sintered ores together by the chemical composition of the sintered raw materials. Since the physical and metallurgical properties of sintered ore can more fully verify the quality of sintered ore, it is particularly important to establish a predictive model of the chemical composition of sintered raw materials on the physical and metallurgical properties of sintered ore. Based on this, this paper uses the GA-RNN model to predict the physical and metallurgical properties of sintered ore by the chemical composition of sintered raw materials, to verify the quality of sintered ore, and to establish the prediction model of the chemical composition of sintered raw materials on the physical and metallurgical properties of sintered ore. It effectively solves the problem of lagging sintering ore test results and can predict the quality of sintered ore before sintering, thus improving the quality of sintered ore, improving efficiency for enterprises, reducing pollutant emissions and protecting the environment.

This paper first constructs an initial sample set of prediction samples and introduces the relationship between the chemical composition of sintered raw materials and the physical and metallurgical properties of sintered ore and the influence of metallurgical performance indicators on the quality of sintered ore. The variable parameters are then analysed theoretically and the input parameters are correlated with the output parameters. Finally, a sinter quality prediction model is developed by predicting the output parameters.

## 2. Prediction Sample Initial Sample Set Construction

### 2.1. Relationship between the Chemical Composition of the Sintering Raw Material and the Physical and Metallurgical Properties of the Sintered Ore

#### 2.1.1. Alkalinity

As the quality of sintered ore depends on its mineral composition, the mineral composition of sintered ore depends on alkalinity. The alkalinity of the sintering process is determined by the SiO_2_ and calcium CaO content, and the strength and metallurgical properties of the sintered ore are closely related to the alkalinity.

#### 2.1.2. MgO

For sintered ore quality, it can also be influenced by the MgO content, which can improve the low-temperature reduction chalking properties of sintered ores. When MgO is sintered, it reacts with Fe_3_O_4_, which will result in the formation of magnesium magnetite, thus inhibiting the oxidation reaction of Fe_3_O_4_, so that the production of the calcium ferrate phase is inhibited, resulting in a reduced reduction of the finished sintered ore and lower cold strength. The addition of MgO when sintering is carried out allows the blast furnace ironmaking slag to be made more fluid, thus allowing its dealkalisation and desulphurisation needs to be met.

#### 2.1.3. TiO_2_

Blast furnace protection requires the addition of a small amount of TiO_2_. However, the presence of TiO_2_ in the sintered ore in the form of the high melting point minerals chalcocite and titania reduces the amount of sintered liquid phase, thus reducing the strength of the sinter.

#### 2.1.4. SiO_2_

SiO_2_ has an important influence on the quality of sintered ore, which forms a large amount of slag phase during sintering, and a study of its slag phase shows that the most abundant substance is SiO_2_. When its content is less than 4.6%, it does not affect the strength of the sintered ore. When its content is higher than 5.3%, it will affect not only the metallurgical properties of the sintered ore but also the strength of the sintered ore.

#### 2.1.5. Al_2_O_3_

For the quality of sintered ore, Al_2_O_3_ plays an important role in influencing it. A certain amount of SiO_2_ and Al_2_O_3_ is essential when sintering if you want the composite calcium ferrate to be produced. Furthermore, if there is no Al_2_O_3_ present, then no calcium complex ferrate is produced during sintering. It is important to clarify that a higher content of Al_2_O_3_ is not better; if its content is higher than 2.0% then it will affect not only the RDI index of the sinter but also the cold strength of the sinter.

### 2.2. Influence of Physical Property Indicators on the Quality of Sintered Ore

The drum index is a physical property index reflecting the mechanical strength of sintered ore. The single determination value is based on the percentage of the total weight of the specimen with a particle size greater than the specified standard after the specimen has been tested in a special drum. The higher the drum index, the higher the mechanical strength of the ore measured [21–23].

(1) $$\begin{array}{c} \text{Drum index }M=\frac{Q\text{ particle size greater than specified standard}}{Q\text{ total specimen weight}}. \end{array}$$

Drum index *M* refers to the ratio of *Q* particle size greater than the specified standard and the total weight of *Q* samples. In (1), *M* refers to the drum index, the unit sign is %; *Q* particle size greater than the specified standard refers to the weight synthesis of sample particle size greater than the specified standard after testing, the unit symbol is T; and the total weight of sample *Q* refers to the total weight of sample, the unit symbol is T.

### 2.3. The Influence of Metallurgical Performance Indexes on the Quality of Sinter

#### 2.3.1. Reducibility of Sinter

The research and analysis of sintered ore can be clear, which refers to the index used to measure the difficulty of combining reducing gas and iron under certain temperature conditions, which can evaluate the quality of ore. Therefore, if the ore has poor reducibility, after being loaded into the blast furnace, it will have an impact on the utilization rate of the upper gas, which will affect its indirect reduction and ultimately affect its output and fuel ratio [24–29]. It is generally believed that RI less than 60% is poorly reducible sinter, and RI more than 80% is good reducible sinter. For sinters with high basicity (*R* = 1.9–2.3), RI > 85% is conventionally required.

#### 2.3.2. Low-Temperature Reduction Powdering Performance

At 400–600 degrees, the crystal lattice changes when the high-valent iron in the sinter is reduced to low-valent iron, and its volume will change at this time. If carbon monoxide is present, then this can lead to carbon precipitation and eventually to cracks and so forth. In this case, the permeability of the column will be affected, as will the porosity and ultimately the production of the blast furnace. Generally, RDI_+3.15_ is required to be greater than 65%. Most sinter production in China is equipped with a certain amount of magnetic fine powder, so RDI_+3.15_ is above 70% [30–33].

#### 2.3.3. Load Softening Performance

The load reduction and softening performance of sinter refers to that after it is loaded into the blast furnace, as the temperature rises and the charge decreases, its reduction reaction continues, which affects the furnace waist and the lower part of the furnace body and finally makes the sinter volume change occur so that it softens. If the alkalinity of the sinter is high, it will begin to soften when the temperature exceeds 1100°C. It should be clear that the softening temperature of the sinter is related to the pore strength of the mineral, and the pore strength is related to the composition of the mineral. When the softening temperature changes, it is affected by the strength of the pore structure. Therefore, it can be considered that the main factor that affects the softening temperature of minerals is the mineral composition [34–36].

## 3. Comparison of LSTM and GA-RNN Algorithms

### 3.1. Long Short-Term Memory Neural Networks

Long Short-Term Memory (LSTM) neural network was proposed by Sepp Hochreiter and Jrgen Schmidhuber in 1997. It is a special kind of RNN, which solves the problem of long-distance dependence arising from the training process of ordinary RNNs, and is built not by traditional neural network nodes but by introducing a “gate” mechanism [37–41].

### 3.2. GA-RNN Prediction Model

#### 3.2.1. Genetic Algorithm Design

The study and analysis of genetic algorithms make it clear that they include not only computer technology but also biological theory. In the field of research, the algorithm is often used to perform simulation studies of biological systems [42, 43]. The algorithm allows the simulation of biological processes, including mutation and selection, and it uses global search and optimization. When evaluating the merit of a solution, the fitness function is used. When rating the source of material, chromosomes are used. In the genetic algorithm, the representative of the solution is the chromosome. When evaluating, the evaluation results can be linked to the genetic probability. By using this approach during continuous iterations, the optimal solution can be obtained. Next, how to optimize the neural network results will be described:

1. First, let the populations initialize. In this step, the population can be allowed to initialize so as to form different RNN populations, taking that population as the first generation.
2. In the second step, the fitness of the population is evaluated. The protection by search finds the smallest individual, and the best structured RNN is represented by that individual.
3. In the third step, genetic manipulation is performed. By using mutations, crossovers, and so forth, new individuals can be produced.
4. In the fourth step, iterative operations are performed. By continuously performing iterations until the end condition appears, there is no uniform guarantee when setting parameters, and although it has been studied by different research scholars, no uniform results have been obtained. In setting the parameters, the article argues that the optimal solution should not be too concentrated, but it should not be too dispersed either. Therefore, the article concentrates the optimal solution near the local solution, so that the significance of its optimal solution appearance is enhanced. The parameters set in the article are as follows: the algebra of termination evolution is 3, the maximum population size is 72, the minimum population size is 6, the maximum mutation is 0.1, the minimum mutation is 0.0001, and the mutation rate is 0.1.

#### 3.2.2. Design of RNN Algorithm

In the article, the RNN algorithm is designed with four chromosomes so that different neural nets can be described. Designing these four chromosomes allows the optimal structure of the search space of the RNN algorithm [44, 45]. The parameters set in the article are as follows: the maximum number of hidden layer neurons is 144, the minimum number of hidden layer neurons is 6, the maximum hidden layers number is 5, and the minimum hidden layers number is 2.

#### 3.2.3. Design of GA-RNN

The article uses genetic algorithm to optimize the RNN neural network to get the optimal initial weights and thresholds, and the initial weights and thresholds of the network are the solutions sought. This allows the weights and thresholds of the RNN to be mapped to population individuals in the genetic algorithm. A set of weights and thresholds correspond to a population of individuals, and the length of an individual is the sum of the number of network weights and thresholds. According to the law of genetic algorithm, the number of individuals of the population and the coding method are appropriately selected to complete the initialization of the population, and then individual fitness values are calculated and selection, crossover, and mutation operations are performed. The most important of these steps is the selection of the fitness function. Since the adjustment of weights and thresholds in RNN is aimed at minimizing the output error of the network, the fitness function can be chosen as the RNN error function in the genetic algorithm, so that the RNN can be combined to complete the optimization of the network. The adaptive functions are as in equations (2) and (3).

Find the fitness function *f*_*fit*_(*f*(*x*)) when the objective function *f*(*x*) is the maximum value:

(2) $$\begin{array}{c} f_{\text{fit}}\left(x\right)=f\left(x\right). \end{array}$$

Find the fitness function *f*_*fit*_(*f*(*x*)) when the objective function *f*(*x*) is minimum as

(3) $$\begin{array}{c} f_{\text{fit}}\left(x\right)=\frac{1}{f\left(x\right)}. \end{array}$$

The calculation is performed by the fitness function, and a large value of individual fitness predicts better results. When individuals have high fitness values, they are better suited to their environment, have a greater chance of being selected, and have a greater probability of passing on good genes to the next generation.

The specific steps of the genetic algorithm to optimize the RNN are as follows:

1. Determine the network topology of RNN. The RNN model generally consists of three parts: hidden layer, output, and input layer.
2. Individual length is used to replace the initial RNN weight threshold length, and the population individuals are coded by genetic algorithm. The coding method is real-valued coding, the population size is set to 60, and the number of generations of genetic evolution is set to 60.
3. Individuals of the population are calculated by equation (2) to obtain the fitness value of each individual, and selection, crossover, and mutation operations are performed, respectively, according to the laws of genetic algorithm.
4. Calculate the fitness value, obtain the optimal weights and thresholds if the conditions are satisfied, and continue the selection, crossover, and variation operations if the conditions are not satisfied.
5. The error is calculated, the weight threshold is updated, the preset requirements are satisfied, the simulation prediction is performed, and the results are obtained. If the condition is not satisfied, continue to calculate the error and update the weight threshold until the condition is satisfied.

**Figure 1:**
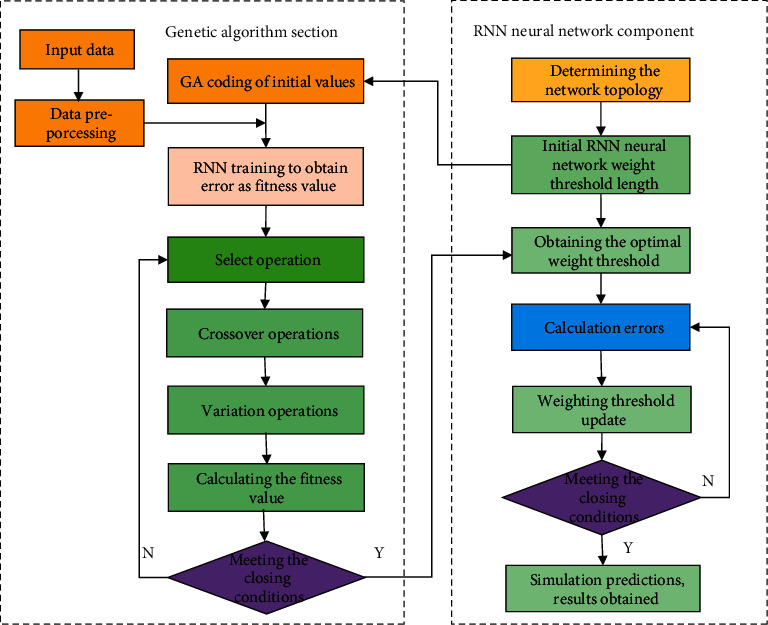
GA-RNN prediction model flowchart.

The GA-RNN prediction model flow chart is shown in Figure 1.

### 3.3. Comparison of LSTM and GA-RNN Algorithms

For the LSTM and GA-RNN algorithms, the mean decision percentage error and goodness-of-fit analyses were performed with the rotating drum index, low-temperature reduction chalking index RDI, reduction degree index RI, load softening temperature T_10%_, and load softening temperature T_40%_, respectively, and the data analyses are shown in Table 1.

**Figure 2:**
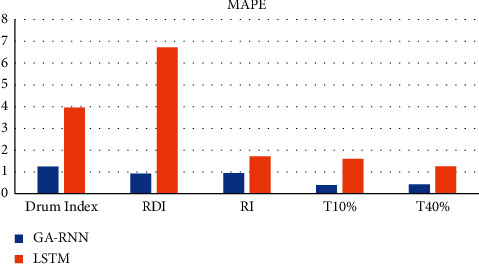
MAPE between GA-RNN and LSTM algorithms.

**Figure 3:**
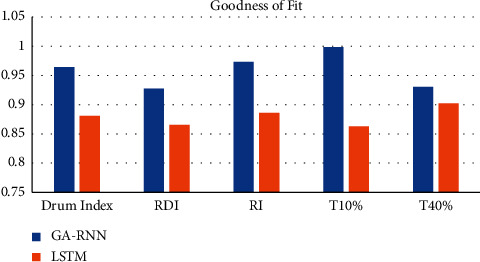
Goodness of fit between GA-RNN and LSTM algorithms.

From Figures 2 and 3, it can be seen that the average decision percentage error of GA-RNN is smaller than that of LSTM algorithm, and GA-RNN has a better fit than LSTM algorithm. Therefore, the GA-RNN algorithm is selected as the prediction algorithm in this paper.

## 4. Theoretical Analysis of Variable Parameters

From research on the sintering process, combined with a lot of practice at the same time, it is clear that the content of iron in the finished product is not significantly different from the content of iron in the mixture. However, due to the large amount of energy lost during the sintering process and the fact that it is calculated using percentages for each chemical composition, when the fuel is consumed, the percentage of iron will also change. A study of silicon and calcium can find that there is no significant change in the percentage of iron.

When constructing the model, it is constructed based on the principle of conservation of materials, and the content of the chemical composition of the raw material of the finished sinter can be determined according to the raw material parameters and the batching parameters, as shown in the following formula:

(4) $$\begin{array}{c} \text{TFe}=\frac{\sum_{i=1}^{n}x_{i}{\text{TFe}}_{i}}{\sum_{i}^{n}x_{i}\left(1-d_{i}\right)},\\ {\text{SiO}}_{2}=\frac{\sum_{i=1}^{n}x_{i}{\text{SiO}}_{2i}}{\sum_{i}^{n}x_{i}\left(1-d_{i}\right)},\\ {\text{Al}}_{2}{\text{O}}_{3}=\frac{\sum_{i=1}^{n}x_{i}{\text{Al}}_{2}{\text{O}}_{3i}}{\sum_{i}^{n}x_{i}\left(1-d_{i}\right)},\\ \text{CaO}=\frac{\sum_{i=1}^{n}x_{i}{\text{CaO}}_{i}}{\sum_{i}^{n}x_{i}\left(1-d_{i}\right)},\\ \text{MgO}=\frac{\sum_{i=1}^{n}x_{i}{\text{MgO}}_{i}}{\sum_{i}^{n}x_{i}\left(1-d_{i}\right)},\\ {\text{TiO}}_{2}=\frac{\sum_{i=1}^{n}x_{i}{\text{TiO}}_{2i}}{\sum_{i}^{n}x_{i}\left(1-d_{i}\right)}. \end{array}$$

In the above formula, CaO, Al_2_O_3_, SiO_2_, TFe, MgO, and TiO_2_ represent the calculated values of sintered calcium oxide content, aluminum oxide content, silicon dioxide content, total iron content, magnesium oxide content, and titanium dioxide content, respectively. The total number of raw material types is represented by the symbol *n*, the burning loss rate is represented by the symbol *d*_*i*_, the iron grade of raw material *i* in the sintered secondary ingredients is denoted by the symbol TFe_i_, the proportion of titanium dioxide content is represented by the symbol TiO_2i_, the content of magnesium oxide is represented by the symbol MgO_i_, the calcium oxide content is indicated by the symbol CaO_i_, the content of aluminum oxide is represented by the symbol Al_2_O_3i_, and the content of silicon dioxide is represented by the symbol SiO_2i_.

The performance indicators of sinter are drum index, RDI_+3.15_, RI, T_10%_, and T_40%_. Among them, drum strength is the physical performance index indicating the mechanical strength of sinter, and RDI_+3.15_ is the low-temperature reduction pulverization index of sinter. RI is the degree of reduction of sintered ore, and T_10%_ and T_40%_ are expressed as load softening performance. The specific calculation method is shown below.

### 4.1. Drum Strength

The parameter of the rotating drum experiment machine is 25 r/min speed, 200 revolutions.

To measure it, the drum index can get the following result:

(5) $$\begin{array}{c} T=\frac{m_{1}}{m_{0}}\times 100\%. \end{array}$$

In the above formula, the mass of the grain size + 6.3 mm after the drum is represented by the symbol m1, and the unit is kg. The mass of the sample entering the drum is indicated by the symbol m0 and the unit is kg.

### 4.2. Determining the Low-Temperature Reduction Pulverization Performance

When measuring this parameter, it is carried out in accordance with the relevant standards of our country. The time for isothermal reduction is 1 h. When it is cooled to room temperature, it can be rotated by a rotating drum for 300 revolutions. Its parameter is Φ130 mm × 200 mm. Then it is sieved with a square hole sieve with different parameters. Its parameters are 0.5 mm, 3.15 mm, and 6.3 mm subsequently expressed by mass percentage when expressing their performance, which are expressed by the symbols RDI_+6.3_, RDI_+3.15_, and RDI_−0.5_, respectively. RDI_+3.15_ is the evaluation index, and RDI_+6.3_ and RDI_−0.5_ are the reference indexes.

The reducing gas component N_2_ is 60%, CO_2_ is 20%, CO is 20%, 500 ± 1 g is the sample weight, the minimum sample size is 10.0 mm, the maximum is not more than 12.5 mm, 15 L/min is the reducing gas flow, the time is 1 h, and 500°C is the reduction temperature.

### 4.3. Measure the Load Softening Performance

When the temperature rises, the softening start temperature is the temperature when the column height shrinks 10%, and the softening end temperature is the temperature when the column height shrinks 40%. The dyeing temperature range is indicated by the symbol △T, and it the temperature difference between the two. 40 mm is the height of the material column, 1 kg/cm^2^ is the load, the minimum sample size is 2 mm, and the maximum is not more than 3 mm.

## 5. Correlation Analysis

In order to explore the correlation between the batching parameters and the properties of sintered ore, the TFe content, SiO_2_ content, Al_2_O_3_ content, CaO content, magnesium oxide MgO content, and titanium dioxide are used. The content of TiO_2_ predicts the drum index, low-temperature reduction pulverization index RDI_+3.15_, reduction index RI, and load softening temperatures T_10%_ and T_40%_ of the sinter, respectively.

Correlation analysis is carried out on the batching parameters of sinters with different batching ratios and related indexes of sinter performance [46–49], and the calculation formula of Pearson correlation coefficient is as follows:

(6) $$\begin{array}{c} \rho_{X,Y}=\frac{\mathrm{cov}\left(X,Y\right)}{\sigma_{X}\sigma_{Y}},\\ \mathrm{cov}\left(X,Y\right)=E\left(\left(X-\mu_{X}\right)\left(Y-\mu_{Y}\right)\right),\\ \sigma_{X}=\sqrt{E\left(X^{2}\right)-E{\left(X\right)}^{2}}. \end{array}$$

In the above formula, *ρ*_*X*,*Y*_ represents the correlation coefficient between *x* and *y*, cov(*X*, *Y*) represents the covariance coefficient between *x* and *y*, *σ*_*X*_ represents the standard deviation of *x*, *E*(*x*) represents the mean value of *x*, Table 2 is the correlation analysis table of the batching parameters and the sinter-related performance indicators (^*∗*^ represents the time; the correlation is significant).

It can be seen from Table 2 that the change of the batching parameters can significantly affect the change of the drum index. The drum index of the sinter is related to the TFe content, SiO_2_ content, and Al_2_O_3_ of the sinter. CaO content and MgO content both have a significant (*P* ≤ 0.05) linear correlation. Among them, TFe content of the sinter is highly negatively correlated with the drum index of the sinter, and the Al_2_O_3_ content has a low degree of negative correlation with the drum index of the sinter and the content of SiO2. There is a low degree of positive correlation with the drum index of sinter, the content of CaO and the drum index of sinter are highly positively correlated, and the content of MgO and the drum index of sinter are moderately positive.

It can be seen from Table 3 that the TFe content and the MgO content of the sinter have a significant linear correlation with the RDI_+3.15_ index of the sinter, and the TFe content is related to the RDI of the sinter. The +3.15 index has a low degree of negative correlation, and the MgO content has a moderately positive correlation with the RDI_+3.15_ index of sinter.

It can be seen from Table 4 that the RI index of sinter has a significant (*P* ≤ 0.05) linear correlation with the TFe content and CaO content in the batching parameters. Among them are the TFe content and the RI of the sinter. The index has a moderately negative correlation, and the CaO content has a moderately positive correlation with the RI index of sintered ore.

It can be seen from Table 5 that there is a significant (*P* ≤ 0.05) linear correlation between the T_10%_ index of sinter and the content of TFe and CaO in the batching parameters. Among them are the content of TFe and the content of sinter. The T_10%_ index has a low degree of positive correlation, and the CaO content has a moderately negative correlation with the T_10%_ index of sinter.

It can be seen from Table 6 that the T_40%_ index of sintered ore has a significant (*P* ≤ 0.05) linear correlation with the TFe content and the Al_2_O_3_ content in the batching parameters. Among them, the content of total iron (TFe) is negatively correlated with the T_40%_ index of sinter, and the content of aluminum oxide (Al_2_O_3_) is negatively correlated with the T_40%_ index of sinter. The T_40%_ index of the ore has a low degree of negative correlation, and the content of Al_2_O_3_ has a low degree of negative correlation with the T_40%_ index of the sinter. In summary, the change of batching parameters can cause changes in the properties of sinter, but this often involves complex mechanisms. There is currently no mature quantitative analysis between batching parameters and sinter performance. The article intends to establish a GA based on GA-RNN model of sinter quality prediction model [50–56] to predict the performance indicators of sinter according to the batching parameters, so as to optimize the production process.

By analysing the process mechanism and characteristics of the sintering process, the input parameters and output parameters of the model were determined. The input parameters are TFe content, SiO_2_ content, Al_2_O_3_ content, CaO content, MgO content, and TiO_2_ content. The output parameters are drum index, reducibility, low-temperature reduction powdering degree, and load softening performance. Finally, based on the experimental data, the correlation between the batching parameters and the performance of the sinter is studied, and it is found that there is a linear relationship between the batching parameters and different performance indicators, which lays the foundation for the establishment of a sinter quality prediction model.

It can be seen that the chemical composition of the sintering raw material is directly related to the physical and metallurgical properties of the sintered ore. Therefore, the GA-RNN model is established to calculate the TFe content and SiO_2_ content of the sintering raw material. TFe content, CaO content, MgO content, Al_2_O_3_ content, and TiO_2_ content are input, and the drum index of sinter, RDI_+3.15_, RI, T_10%_, and T_40%_ are output; a quality prediction model for sintered ore is established to effectively predict the quality of sintered ore, which can greatly reduce the failure rate of sintering, effectively save energy, and improve production quality.

**Figure 4:**
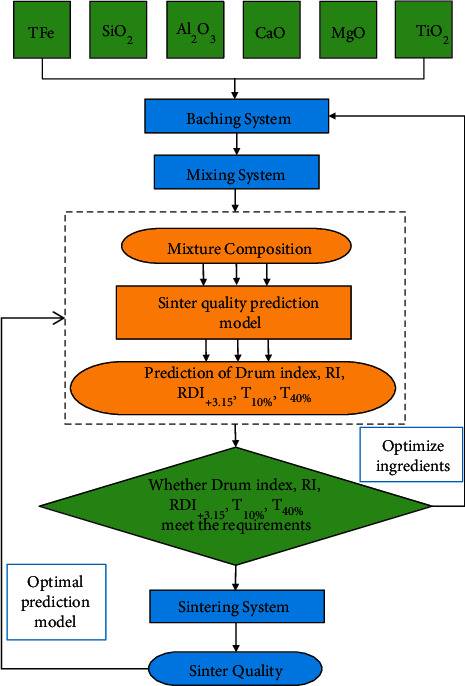
GA-RNN sinter-based quality prediction model.

Therefore, the establishment of a GA-RNN sinter-based quality prediction model is shown in Figure 4.

## 6. Model Prediction of Sinter Quality

Use the constructed network model to calculate the absolute value of the error of the test sample, and obtain the average error, as shown in equation (7). Use Matlab to perform RNN network predictions and obtain prediction errors.

(7) $$\begin{array}{c} e=\frac{1}{n}\sum_{i=1}^{n}\left|\frac{\hat{y}\left(x_{i}\right)-y\left(x_{i}\right)}{y\left(x_{i}\right)}\right|\times 100\%. \end{array}$$

In the above formula, $\overset{\wedge }{y}\left(x_{i}\right)$ is the predicted value of the output parameter; *y*(*x*_*i*_) is the real value of the output parameter; *n* is the number of test samples.

### 6.1. Prediction of Drum Index

**Figure 5:**
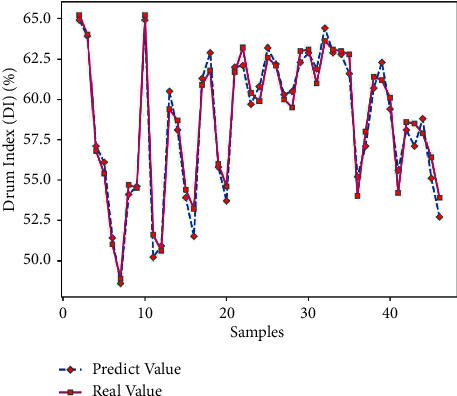
Drum index prediction output.

**Figure 6:**
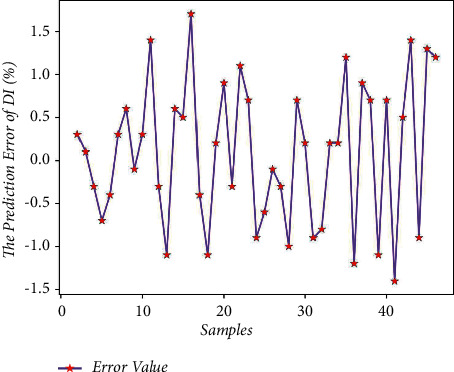
Drum index prediction error.

There are 150 sets of data samples, of which 70% are used for training and 30% are used for testing. Take the first 105 sets of data from the data samples for model training, and the last 45 sets of data for model testing. Using Matlab to perform RNN network prediction to obtain the predicted value of the drum index of 45 sets of test data, the comparison of the results is shown in Table 7. The average prediction error of the drum index obtained by formula (7) is 1.24%, which is relatively small; and get the predicted output results of Figures 5 and 6.

**Figure 7:**
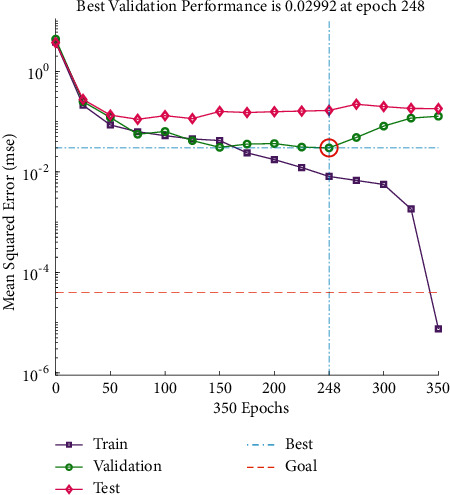
Drum index iteration diagram.

It can be seen from Figures 5 and 6 that there is a little difference between the predicted value of the drum index and the real value curve, the change trend of the two is the same, and the prediction error fluctuates within the range of ±2. From Figure 7, it can be seen that the number of iterations is 248. When the minimum mean square error (MSE) is 0.02992 and the prediction effect of the network is better, it is indicated that the prediction of the sinter drum index can be achieved from the composition of the sinter raw material.

### 6.2. Prediction of Low-Temperature Reduction Powdering Index (RDI)

**Figure 8:**
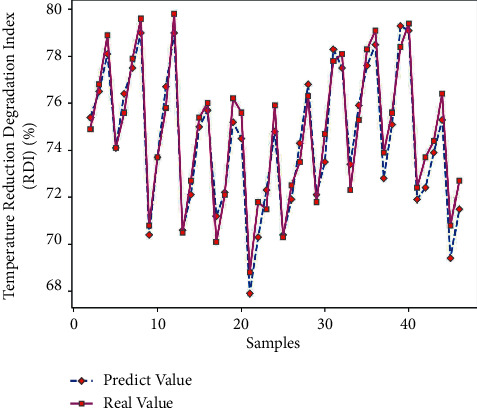
Schematic diagram of RDI output.

**Figure 9:**
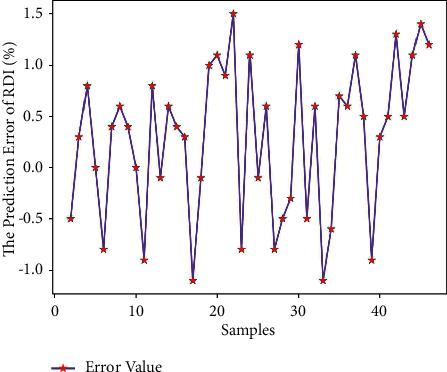
RDI error diagram.

Use Matlab to perform RNN network prediction to obtain the low-temperature reduction pulverization index (RDI) prediction value of 45 sets of test data. The comparison of the results is shown in Table 8. By formula (7), the average prediction error of the low-temperature reduction pulverization index RDI is 0.92%, which is relatively small; and get the predicted output results of Figures 8 and 9.

**Figure 10:**
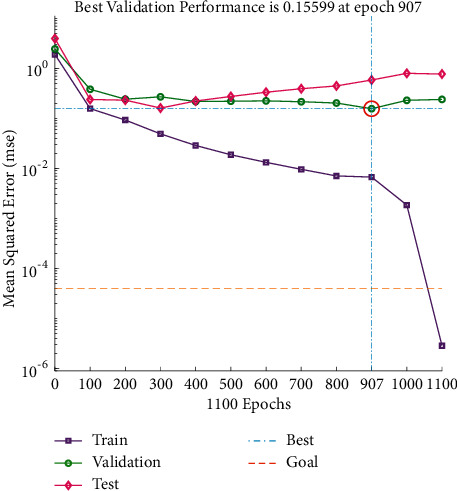
The RDI iteration figure.

It can be seen from Figures 8 and 9 that the predicted value of the low-temperature reduction powder index RDI and the real value curve have a small difference, the change trend of the two is the same, and the prediction error fluctuates within the range of ±1.5. From Figure 10, it can be seen that when the number of iterations is 907, the minimum mean square error (MSE) is 0.15599, and the prediction effect of the network is better, indicating that the prediction of the low-temperature reduction pulverization index RDI of the sinter can be achieved from the theoretical composition of the sinter.

### 6.3. Prediction of Reduction Index (RI)

**Figure 11:**
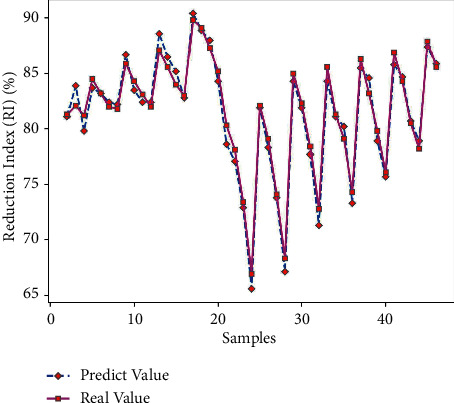
RI predictive output.

**Figure 12:**
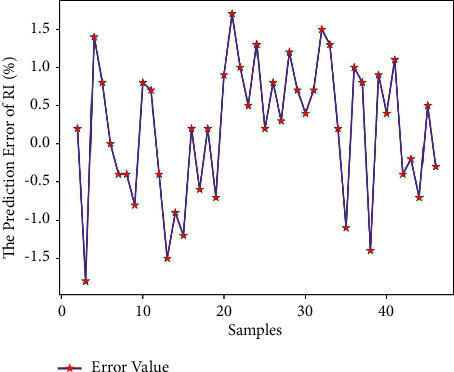
RI prediction error.

Use Matlab to perform RNN network prediction to obtain the predicted value of the reduction index (RI) of 45 sets of test data. The comparison of the results is shown in Table 9. The average prediction error of the reduction index (RI) obtained by formula (7) is 0.95%, which is relatively small; and get the predicted output results of Figures 11 and 12.

**Figure 13:**
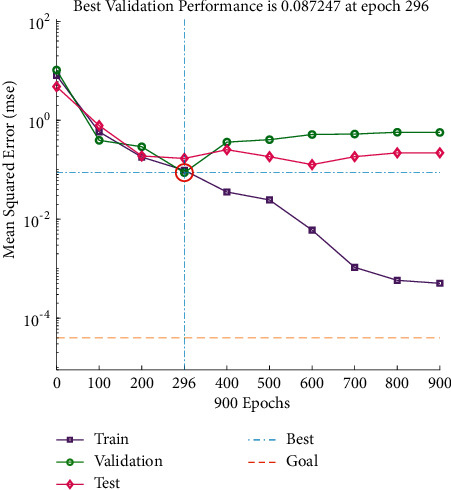
RI iterative figure.

It can be seen from Figures 11 and 12 that the predicted value of the reduction index (RI) has a small difference with the real value curve, the change trend of the two is the same, and the prediction error fluctuates within the range of ±2. From Figure 13, it can be seen that when the number of iterations is 296, the minimum mean square error (MSE) obtained is 0.087247, and the prediction effect of the network is better, indicating that the theoretical composition of the sinter can better predict the reduction index (RI) of the sinter.

### 6.4. Prediction of Load Softening Temperature T_10%_

**Figure 14:**
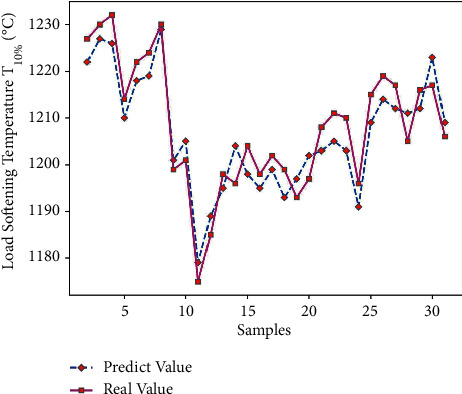
Load softening temperature T_10%_ predicted output.

**Figure 15:**
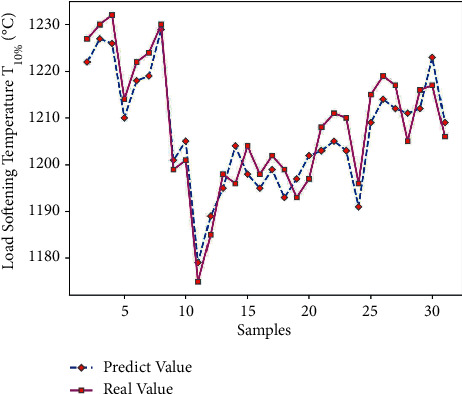
T_10%_ prediction error of load softening temperature.

Using Matlab to perform RNN network prediction to obtain the predicted value of load softening temperature T_10%_ of 45 sets of test data, the results are compared in Table 10. Through formula (7), the average prediction error of load softening temperature T_10%_ is 0.40%, which is relatively small; and get the predicted output results of Figures 14 and 15.

**Figure 16:**
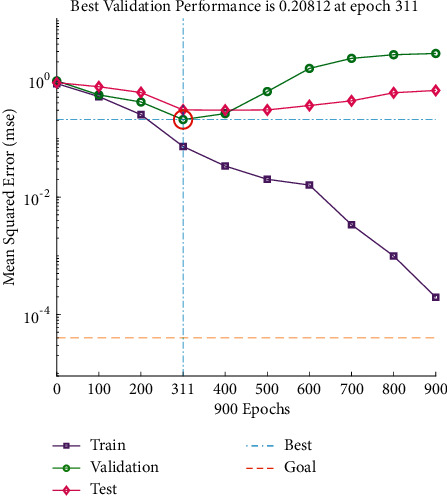
T_10%_ iteration diagram of load softening temperature.

It can be seen from Figures 14 and 15 that the predicted value of load softening temperature T_10%_ has a small difference with the real value curve, the change trend of the two is the same, and the prediction error fluctuates within the range of ±10; because the temperature base is 1000°C above, the prediction error is relatively small.  Figure 16 shows that when the number of iterations is 311, the minimum mean square error (MSE) is 0.20812, and the prediction effect of the network is better, indicating that the theoretical composition of the sinter can better predict the T_10%_ softening temperature of sinter under load.

### 6.5. Prediction of Load Softening Temperature T_40%_

**Figure 17:**
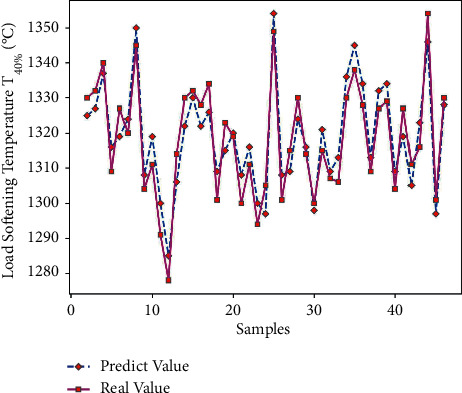
Load softening temperature T_40%_ predicted output.

**Figure 18:**
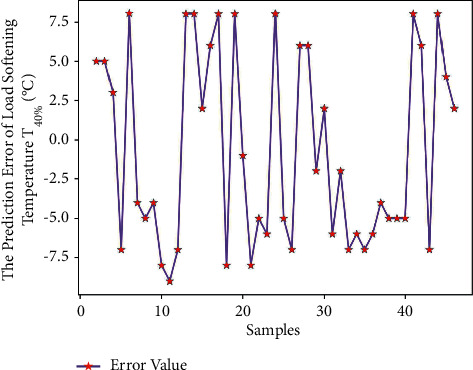
T_40%_ prediction error of load softening temperature.

Use Matlab to perform RNN network prediction to obtain the predicted value of load softening temperature T_40%_ of 45 sets of test data. The comparison of the results is shown in Table 11. Through formula (7), the average prediction error of load softening temperature T_40%_ is 0.43%, which is relatively small; and get the predicted output results of Figures 17 and 18.

**Figure 19:**
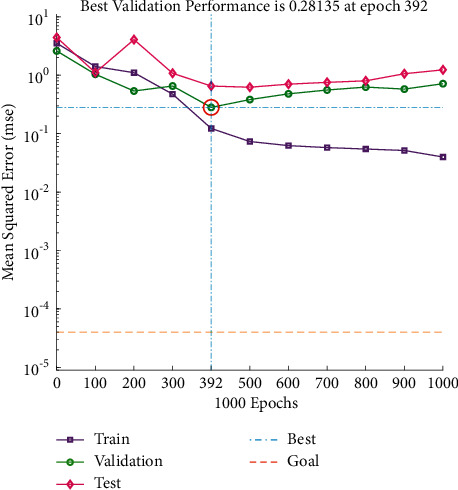
T_40%_ iteration diagram of load softening temperature.

It can be seen from Figures 17 and 18 that the predicted value of the load softening temperature T_40%_ and the real value curve have a small difference, the change trend of the two is the same, and the prediction error is floating within the range of ±10; since the temperatures are above 1000°C, the prediction error is relatively small.  Figure 19 shows that when the number of iterations is 392, the minimum mean square error (MSE) is 0.28135, and the prediction effect of the network is better, indicating that the theoretical composition of the sinter can better predict the T_40%_ softening temperature of sinter under load.

## 7. Conclusion

In view of the large time lag in the detection of sinter, the article verifies the relationship between the chemical composition of the sintering raw material and the physical and metallurgical properties of the sinter through correlation analysis. This led to the determination of a GA-RNN quality prediction model with total iron (TFe) content, silicon dioxide (SiO_2_) content, aluminium oxide (Al_2_O_3_) content, calcium oxide (CaO) content, magnesium oxide (MgO) content, and titanium dioxide (TiO_2_) content as input parameters and the sinter drum index, low-temperature reduction pulverization index (RDI_+3.15_), reduction index (RI), and load softening temperatures T_10%_ and T_40%_ as the output parameters of the GA-RNN quality prediction model. The GA-RNN algorithm is used to predict the five quality indicators of sinter. The average prediction error of the drum index is 1.24%, the average prediction error of the low-temperature reduction pulverization index (RDI) is 0.92%, the average prediction error of the reduction index (RI) is 0.95%, the average prediction error of the load softening temperature T_10%_ is 0.40%, and the average prediction error of the load softening temperature T_40%_ is 0.43%, and all are within the operating time threshold. The establishment of this model can accurately predict the physical properties and metallurgical properties of the sinter through the chemical composition of the sintering raw materials before sintering, so as to predict the quality of the sinter. It can save costs for enterprises and can save energy, prevent environmental pollution, and protect the environment.

## Figures and Tables

**Table 1:** Comparison of GA-RNN and LSTM algorithms.

Indicators	Algorithm	MAPE	*R* ^2^

Drum index	GA-RNN	1.24	0.9642
	LSTM	3.96	0.8807

RDI	GA-RNN	0.92	0.9273
	LSTM	6.72	0.8656

RI	GA-RNN	0.95	0.973
	LSTM	1.71	0.886

T_10%_	GA-RNN	0.4	0.9985
	LSTM	1.6	0.8627

T_40%_	GA-RNN	0.43	0.9304
	LSTM	1.26	0.9023

**Table 2:** Correlation analysis table of batching parameters and drum index.

	TFe	SiO_2_	Al_2_O_3_	CaO	MgO	TiO_2_
Pearson	−0.704^*∗∗*^	0.294^*∗*^	−0.262^*∗*^	0.858^*∗∗*^	0.592^*∗∗*^	0.011
*P*	0.000	0.018	0.037	0.000	0.000	0.930

**Table 3:** Correlation analysis table of ingredient parameters and RDI_+3.15_.

	TFe	SiO_2_	Al_2_O_3_	CaO	MgO	TiO_2_
Pearson	−0.339^*∗∗*^	−0.094	0.021	0.019	0.592^*∗∗*^	0.011
*P*	0.006	0.462	0.868	0.879	0.000	0.930

**Table 4:** Correlation analysis table of ingredient parameters and RI.

	TFe	SiO_2_	Al_2_O_3_	CaO	MgO	TiO_2_
Pearson	−0.528^*∗∗*^	0.107	−0.187	0.668^*∗∗*^	−0.060	−0.070
*P*	0.000	0.400	0.140	0.000	0.636	0.581

**Table 5:** Correlation analysis table of ingredient parameters and T_10%_.

	TFe	SiO_2_	Al_2_O_3_	CaO	MgO	TiO_2_
Pearson	0.305^*∗*^	−0.156	0.058	−0.472^*∗∗*^	0.161	0.105
*P*	0.014	0.218	0.650	0.000	0.205	0.410

**Table 6:** Correlation analysis table of ingredient parameters and T_40%_.

	TFe	SiO_2_	Al_2_O_3_	CaO	MgO	TiO_2_
Pearson	−0.245	−0.135	−0.326^*∗∗*^	0.115	0.234	−0.068
*P*	0.051	0.289	0.009	0.366	0.063	0.591

**Table 7:** Comparison between predicted value and real value of drum index.

Group	Predicted value $\overset{\wedge }{y}\left(x_{i}\right)/\%$	Real value *y*(*x*_*i*_)/%	Group	Predicted value $\overset{\wedge }{y}\left(x_{i}\right)/\%$	Real value *y*(*x*_*i*_)/%	Group	Predicted value $\overset{\wedge }{y}\left(x_{i}\right)/\%$	Real value *y*(*x*_*i*_)/%

1	64.9	65.2	16	61.3	60.9	31	64.4	63.6
2	63.9	64	17	62.9	61.8	32	62.9	63.1
3	57.1	56.8	18	55.8	56	33	62.8	63
4	56.1	55.4	19	53.7	54.6	34	61.6	62.8
5	51.4	51	20	62.0	61.7	35	55.2	54
6	48.6	48.9	21	62.1	63.2	36	57.1	58
7	54.1	54.7	22	59.7	60.4	37	60.7	61.4
8	54.6	54.5	23	60.8	59.9	38	62.3	61.2
9	64.9	65.2	24	63.2	62.6	39	59.4	60.1
10	50.2	51.6	25	62.2	62.1	40	55.6	54.2
11	50.9	50.6	26	60.3	60	41	58.1	58.6
12	60.5	59.4	27	60.5	59.5	42	57.1	58.5
13	58.1	58.7	28	62.3	63	43	58.8	57.9
14	53.9	54.4	29	62.9	63.1	44	55.1	56.4
15	51.5	53.2	30	61.9	61	45	52.7	53.9

**Table 8:** the predicted value and the real value of RDI.

Group	Predicted value $\overset{\wedge }{y}\left(x_{i}\right)/\%$	Real value *y*(*x*_*i*_)/%	Group	Predicted value $\overset{\wedge }{y}\left(x_{i}\right)/\%$	Real value *y*(*x*_*i*_)/%	Group	Predicted value $\overset{\wedge }{y}\left(x_{i}\right)/\%$	Real value *y*(*x*_*i*_)/%
1	75.4	74.9	16	71.2	70.1	31	77.5	78.1
2	76.5	76.8	17	72.2	72.1	32	73.4	72.3
3	78.1	78.9	18	75.2	76.2	33	75.9	75.3
4	74.1	74.1	19	74.5	75.6	34	77.6	78.3
5	76.4	75.6	20	67.9	68.8	35	78.5	79.1
6	77.5	77.9	21	70.3	71.8	36	72.8	73.9
7	79.0	79.6	22	72.3	71.5	37	75.1	75.6
8	70.4	70.8	23	74.8	75.9	38	79.3	78.4
9	73.7	73.7	24	70.4	70.3	39	79.1	79.4
10	76.7	75.8	25	71.9	72.5	40	71.9	72.4
11	79.0	79.8	26	74.3	73.5	41	72.4	73.7
12	70.6	70.5	27	76.8	76.3	42	73.9	74.4
13	72.1	72.7	28	72.1	71.8	43	75.3	76.4
14	75.0	75.4	29	73.5	74.7	44	69.4	70.8
15	75.7	76	30	78.3	77.8	45	71.5	72.7

**Table 9:** predicted value and the real value of RI.

Group	Predicted value $\overset{\wedge }{y}\left(x_{i}\right)/\%$	Real value *y*(*x*_*i*_)/%	Group	Predicted value $\overset{\wedge }{y}\left(x_{i}\right)/\%$	Real value *y*(*x*_*i*_)/%	Group	Predicted value $\overset{\wedge }{y}\left(x_{i}\right)/\%$	Real value *y*(*x*_*i*_)/%
1	81.1	81.3	16	90.4	89.8	31	71.3	72.8
2	83.9	82.1	17	88.9	89.1	32	84.3	85.6
3	79.8	81.2	18	88.0	87.3	33	81.1	81.3
4	83.7	84.5	19	84.3	85.2	34	80.2	79.1
5	83.2	83.2	20	78.6	80.3	35	73.3	74.3
6	82.4	82	21	77.1	78.1	36	85.5	86.3
7	82.2	81.8	22	72.9	73.4	37	84.6	83.2
8	86.7	85.9	23	65.6	66.9	38	78.9	79.8
9	83.5	84.3	24	81.9	82.1	39	75.7	76.1
10	82.4	83.1	25	78.3	79.1	40	85.8	86.9
11	82.4	82	26	73.8	74.1	41	84.7	84.3
12	88.6	87.1	27	67.1	68.3	42	80.7	80.5
13	86.5	85.6	28	84.3	85	43	78.9	78.2
14	85.2	84	29	81.9	82.3	44	87.4	87.9
15	82.8	83	30	77.7	78.4	45	85.9	85.6

**Table 10:** Comparison between the predicted value and the actual value of T_10%_ softening temperature under load.

Group	Predicted value $\overset{\wedge }{y}\left(x_{i}\right)/\%$	Real value *y*(*x*_*i*_)/%	Group	Predicted value $\overset{\wedge }{y}\left(x_{i}\right)/\%$	Real value *y*(*x*_*i*_)/%	Group	Predicted value $\overset{\wedge }{y}\left(x_{i}\right)/\%$	Real value *y*(*x*_*i*_)/%

1	1222	1227	16	1199	1202	31	1202	1200
2	1227	1230	17	1193	1199	32	1199	1201
3	1226	1232	18	1197	1193	33	1193	1227
4	1210	1214	19	1202	1197	34	1197	1215
5	1218	1222	20	1203	1208	35	1208	1204
6	1219	1224	21	1205	1211	36	1211	1205
7	1229	1230	22	1203	1210	37	1210	1222
8	1201	1199	23	1191	1196	38	1196	1212
9	1205	1201	24	1209	1215	39	1215	1206
10	1179	1175	25	1214	1219	40	1219	1202
11	1189	1185	26	1212	1217	41	1217	1201
12	1195	1198	27	1211	1205	42	1205	1201
13	1204	1196	28	1212	1216	43	1216	1219
14	1198	1204	29	1223	1217	44	1217	1198
15	1195	1198	30	1209	1206	45	1206	1196

**Table 11:** Comparison between the predicted value and the actual value of T_40%_ softening temperature under load.

Group	Predicted value $\overset{\wedge }{y}\left(x_{i}\right)/\%$	Real value *y*(*x*_*i*_)/%	Group	Predicted value $\overset{\wedge }{y}\left(x_{i}\right)/\%$	Real value *y*(*x*_*i*_)/%	Group	Predicted value $\overset{\wedge }{y}\left(x_{i}\right)/\%$	Real value *y*(*x*_*i*_)/%

1	1325	1330	16	1326	1334	31	1309	1307
2	1327	1332	17	1309	1301	32	1313	1306
3	1337	1340	18	1315	1323	33	1336	1330
4	1316	1309	19	1320	1319	34	1345	1338
5	1319	1327	20	1308	1300	35	1334	1328
6	1324	1320	21	1316	1311	36	1313	1309
7	1350	1345	22	1300	1294	37	1332	1327
8	1308	1304	23	1297	1305	38	1334	1329
9	1319	1311	24	1354	1349	39	1309	1304
10	1300	1291	25	1308	1301	40	1319	1327
11	1285	1278	26	1309	1315	41	1305	1311
12	1306	1314	27	1324	1330	42	1323	1316
13	1322	1330	28	1316	1314	43	1346	1354
14	1330	1332	29	1298	1300	44	1297	1301
15	1322	1328	30	1321	1315	45	1328	1330

## Data Availability

The data used to support the findings of this study are available from the corresponding author upon request.
